# Prevalence and Risk Factors of Human Intestinal Parasites in Roudehen, Tehran Province, Iran

**Published:** 2017

**Authors:** Nasrin HEMMATI, Elham RAZMJOU, Saeideh HASHEMI-HAFSHEJANI, Abbas MOTEVALIAN, Lameh AKHLAGHI, Ahmad Reza MEAMAR

**Affiliations:** 1. Dept. of Parasitology and Mycology, School of Medicine, Iran University of Medical Sciences, Tehran, Iran; 2. Dept. of Epidemiology, School of Public Health, Iran University of Medical Sciences, Tehran, Iran

**Keywords:** *Blastocystis*, *Entamoeba*, Intestinal parasite, Prevalence, Protozoa

## Abstract

**Background:**

Intestinal parasitic infections are among the most common infections and health problems worldwide. Due to the lack of epidemiologic information of such infections, the prevalence of, and the risk factors for, enteric parasites were investigated in residents of Roudehen, Tehran Province, Iran.

**Methods:**

In this cross-sectional study, 561 triple fecal samples were collected through a two-stage cluster-sampling protocol from Jun to Dec 2014. The samples were examined by formalin-ether concentration, culture, and with molecular methods.

**Results:**

The prevalence of enteric parasites was 32.7% (95% CI 27.3–38). *Blastocystis* sp. was the most common intestinal protozoan (28.4%; 95% CI 23.7–33.0). The formalin-ether concentration and culture methods detected *Blastocystis* sp., *Entamoeba coli*, *Giardia intestinalis*, *Dientamoeba fragilis*, *Iodamoeba butschlii*, *Entamoeba complex cysts or trophozoite*, *Chilomastix mesnilii*, and *Enterobius vermicularis*. Single-round PCR assay for *Entamoeba* complex were identified *Entamoeba dispar* and *E. moshkovskii*. *E. histolytica* was not observed in any specimen. Multivariate analysis showed a significant association of parasites with water source and close animal contact. There was no correlation between infections and gender, age, occupation, education, or travel history. Protozoan infections were more common than helminth infections.

**Conclusion:**

This study revealed a high prevalence of enteric protozoan parasite infection among citizens of Rodehen. As most of the species detected are transmitted through a water-resistant cyst, public and individual education on personal hygiene should be considered to reduce transmission of intestinal parasites in the population.

## Introduction

Intestinal parasite infection remains a health problem, especially in developing countries, in spite of efforts by the WHO and governments to eliminate parasites and prevent and treat parasitic disease. Worldwide, over 3.5 billion people are infected with an intestinal parasite annually, and 4.5 million exhibit clinical symptoms (1). Parasitic infections may be asymptomatic or extend to morbidity and mortality, depending on the nutrition and health status of affected individuals (2, 3).

The prevalence of intestinal parasitic infections has been related to low levels of education, health practices, malnourishment, contaminated food and/or water, climate, population growth, and socioeconomic and health conditions as well as close contact with animals (2–5). Intestinal parasitic diseases are among the most important infectious diseases that have a direct relationship to personal and public hygiene (4, 5).

A dramatic decrease in the prevalence of parasitic infections has been reported in Iran (6), although the occurrence of enteric protozoans is still common and remain a health problem in some parts of the country (4, 7–9). Prior studies reported *Giardia intestinalis* and *Entamoeba coli* to be the most common intestinal parasites in Iran (8, 9), but recently *Blastocystis* sp. has been suggested to be the most prevalent (10, 11).

The goal of this study was to determine the prevalence of, and the risk factors for, intestinal parasites in Roudehen, Tehran Province, Iran.

## Materials and Methods

### Study Area

Roudehen is a city in Damavand County, Tehran Province; northern Iran located 35 km east of Tehran, in the Alborz Mountains. Roudehan has a mountain temperate climate. It is 1850 m above sea level and has an area of 50 km^2^ with a population of 21477 in 7393 households (12).

### Study design and sampling

The cross-sectional study was conducted from Jun to Dec 2014. The triple faecal samples from 561 residents were collected in a two-stage cluster-sampling scheme. In the first stage, the city was divided into 82 blocks, and 23 blocks were randomly selected. In the second stage, 10 households within each block were randomly selected. All members of selected households were invited to participate in the study. If in the selected household, no one answered to investigators on two separate dates, or if all individuals in the household refused to participate in the study, the next household was invited.

The aim and design of the study were explained to household members. Agreeable individuals and the parents of children under age 15 gave written consent and filled a questionnaire form including sex, age, occupation, education, drinking water source, and history of travel and animal contact. Three samples were collected from each participant with one-day interval.

### Laboratory procedures

Stool samples were transferred immediately after collection to the Department of Parasitology and Mycology, Iran University of Medical Sciences, for examination and identification of parasites.

### Parasite examination

Pea-sized pieces of fecal samples were submitted to formalin-ether concentration technique to identify ova, cysts or oocysts of parasites. The remaining sample was emulsified in PBS and passed through a two-layer gauze filter to remove larger particulates, and the suspension was centrifuged at 1000 × g for 5 min. The sediments were separated into separate portions for culture and molecular study. One hundred mg of the sediment was resuspended in 200 μl 2% polyvinylpolypyrrolidone in PBS and stored at −80 °C until submitted to DNA extraction and 500 μl of which was cultured in a biphasic medium, horse serum slant overlaid with 5 ml of Ringer’s solution, 200 μl of rice starch (5 mg/ml) and penicillin-streptomycin incubated at 35.5 °C and examined three times at 48 h intervals (13). Samples were considered positive if helminth eggs, larvae, or cysts, and/or trophozoites of protozoans were detected by at least one of the two techniques.

### Molecular examination

All samples containing *Entamoeba* trophozoites or cysts were submitted to molecular study. A single-round PCR assay (14) was performed for discrimination of *Entamoeba* species (*Entamoeba dispar, Entamoeba moshkovskii, Entamoeba histolytica*). DNA was extracted from 200 μl of frozen sediment of stool samples using QIAamp DNA minikit (Qiagen, Hilden, Germany) according to manufacturer’s instruction. PCR of *Entamoeba* complex was performed using the forward primer EntaF and reverse primers EhR, EdR, and EmR, specific for *E. histolytica*, *E. dispar*, and *E. moshkovskii*, respectively (14). The PCR reaction was designed to amplify a 166 bp PCR product with *E. histolytica* DNA, a 752-bp PCR product with *E. dispar* DNA, and a 580 bp product with *E. moshkovskii* DNA of a small-subunit rRNA gene (14). In the PCR reaction, a 25 μl PCR reaction mixture consisted of 2 μl of template DNA, 0.25 μM of each the forward primer EntaF and reverse primers EhR, EdR, and EmR primers, 12.5 μl Taq DNA polymerase mastermix (Ampliqon, Denmark), and 6.5 μl H_2_O. PCR was carried out with the following amplification conditions: 1 cycle at 94 °C for 3 min followed by 30 cycles at 94 °C for 60 sec, 58 °C for 60 sec, and 72 °C for 60 sec, and a final extension of 72 °C for 7 minutes. Sterile distilled water was included as a negative control and the mix of genomic DNA of *E. histolytica* reference strain HM-1, IMSS Clone 6 (ATCC® Number, 50527 ™), *E. dispar* reference strain SAW 760 (ATCC® Number, PRA260™), and *E. moshkovskii* reference strain Laredo (ATCC® Number, 30042 ™) were used, as positive control, to validate the results of multiplex PCR. The PCR products were detected on ethidium bromide stained 1.5% agarose gels.

### Data management and analysis

The sampling weights were calculated by multiplication of three weighting components: 1) inverse sampling weight, 2) inverse of nonresponse for each block, and 3) post-stratification weights calculated by dividing census-derived population weights by sample weights for each of 18 age/sex groups (Table 1).

**Table 1:** Socio-demographic characteristics of residents of Roudehen, Tehran Province, Iran from Jun to Dec 2014

***Characteristics***	***n***	***Un-weighted %***	***Weighted %***
Sex			
Female	324	57.8	49.7
Male	237	42.3	50.3
Age group			
0–10	111	19.8	15.6
11–20	72	12.8	14.1
21–30	84	15.0	21.6
31–40	105	18.7	20.0
41–50	82	14.6	13.5
51–60	62	11.1	8.1
60<	45	8.0	7.1
Occupation			
Housewife	191	34.1	27.5
Student	138	24.6	25.1
Employed	132	23.5	30.6
Children	61	10.9	9.1
Retired	24	4.3	4.5
Unemployed	15	2.7	3.2
Education			
Illiterate	31	5.5	4.0
First elementary school	133	23.7	20.7
Second elementary school	80	14.3	12.4
High school	145	25.9	29.2
University	111	19.8	24.6
Children under 6-yr	61	10.9	9.1
Water Source			
Tap Water			
Yes	464	82.7	83.2
No	97	17.3	16.8
Spring water			
Yes	58	10.3	11.1
No	503	89.7	88.9
Filtered water			
Yes	50	8.9	8.8
No	511	91.1	91.2
Travel history			
Yes	338	60.3	59.7
No	223	39.8	40.4
Animal contact			
Yes	72	12.8	13.3
No	489	87.2	86.7

For descriptive analysis, frequency and percentage rates were used to describe characteristics of the studied population, including the prevalence of intestinal parasites. All variables were included in multiple logistic regression analyses to identify the adjusted odds ratios (aOR) of risk factors for intestinal parasite infection. Stata v.10.0 (StataCorp LP, Texas, USA) was used to conduct statistical procedures.

### Ethical considerations and treatment

The study protocol was reviewed and approved by the Ethics Committee of Iran University of Medical Sciences (IUMS) with the code number: IR.IUMS.REC 93-04-30-25381, following the revised Helsinki Declaration of 2008. Each participant was asked to sign an informed consent. For children under 15 yr, one parent was required to agree and sign the consent form. At the end of the study, a laboratory report was provided to each participant, and, if indicated, they were referred for medical care.

## Results

### Socio-demographic characteristics of the study participants

Two-hundred-thirty of 906 approached households agreed to participate in the study, for a response rate of approximately 25.4%. Of the 832 individuals in these households, 561 (67.4%) completed participation in the investigation. Over six months, triple fecal samples from 561 subjects, including 324 (50.3%) females and 237 (49.7%) males from four months to 90 yr of age (mean 31 yr) were obtained (Table 1).

### Prevalence of intestinal parasites

At least one species of intestinal parasite was found in 32.7% (95% CI 27.3–38.0) of residents [31.4% (95% CI 25.7–37.0) of females and 33.9% (95% CI 24.7–43.9) of males] (Table 2). *Blastocystis* sp. (28.4%; 95% CI 23.7–33.0) was the most common intestinal parasite observed.

**Table 2:** Prevalence of intestinal parasites in residents of Roudehen, Tehran, Iran from Jun to Dec 2014

**Parasite**	**Male (n = 237)**	**Female (n = 324)**	**Total (n = 561)**	**95% CI^a^**
	n (%)	n (%)	n (%)	
*Blastocystis* sp.	64 (27.5)	95 (29.4)	159 (28.4)	23.7–33.0
*Entamoeba* coli	13 (6.3)	15 (5.2)	28 (5.8)	3.5–8.0
*Giardia intestinalis*	5 (2.0)	2 (0.4)	7 (1.2)	0.4–2.0
*Entamoeba dispar*	0 (0)	2 (0.4)	2 (0.4)	0–1.1
*Entamoeba moshkovskii*	0 (0)	1 (0.2)	1 (0.2)	0–0.4
*Chilomastix mesnili*	1 (0.4)	1 (0.2)	2 (0.3)	0–0.7
*Iodamoeba butschlii*	2 (0.7)	2 (0.6)	4 (0.7)	0–1.5
*Dientamoeba fragilis*	3 (1.3)	3 (0.9)	6 (1.1)	0.2–2.0
*Enterobius vermicularis*	1 (0.2)	0 (0)	1 (0.2)	0–0.4
Total	78 (33.9)	102 (31.4)	180 (32.7)	27.3–38.0

a CI: confidence interval

Of 180 (32.7%) infected individuals, 159 (14.2%) were infected with a single species, 20 (1.4%) with two species, and four (0.5%) with three species.

The formalin-ether concentration detected 115 (20.5%) *Blastocystis* sp., 25 (5.3%) *Entamoeba coli*, seven (1.2%) *Giardia intestinalis*, four (0.7%) *Iodamoeba butschlii*, one (0.2%) *Chilomastix mesnili*, one (0.2%) four-nuclei *Entamoeba* cysts, and one (0.2%) *Enterobius vermicularis*.

Culture revealed *Blastocystis* sp. in 115 (20.3%), *E. coli* in 13 (2.5%), *Dientamoeba fragilis* in six (1.1%), *I. butschlii* in two (0.3%), *Entamoeba* complex trophozoite in two (0.4%), and *C. mesnili* in two (0.3%) of the population (Table 2).

**Fig. 1:**
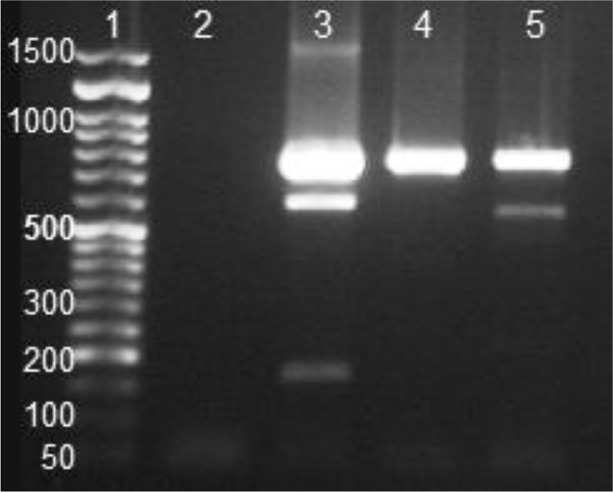
Single-Round PCR amplification of DNA extracted from fecal of *Entamoeba* complex positive samples. Lane 1, 50 bp ladder (Cat NO. PR901633); Lane 2, negative control; Lane 3, positive control: a mixture of standard DNA of *E. histolytica* (166 bp), *E. dispar* (752 bp), and *E. moshkovskii* (580 bp); lane 4–5 two cyst positive samples; line 4, 752 bp amplification showed *E. dispar*-positive infection; line 5, 580 and 752 bp amplified products showed co-infection of *E. moshkovskii* and *E. dispar.*

*Entamoeba* complex positive samples were identified by single-round PCR assay in two samples: one was *E. dispar,* and the other sample showed mixed infection of *E. dispar* and *E. moshkovskii*. *Entamoeba histolytica* was not observed in any specimen (Fig. 1).

### Intestinal parasite and possible risk factors

The results of unadjusted and adjusted logistic regression analyses of the socio-demographic correlates of intestinal parasitic infections among residents of Roudehen are presented in Table 3.

**Table 3:** Univariate and multivariate analysis of intestinal parasitic infections and potential risk factors in residents of Roudehen, Tehran Province, Iran from Jun to Dec 2014

**Risk factor**	**Prevalence % (95 % CI^a^)**	**OR^b^ (95 % CI)**	**aOR^c^ (95 % CI)**
Sex			
Female	31.4 (25.7–37.0)	1	1
Male	33.9 (24.7–43.9)	1.1 (0.7–1.8)	1.4 (0.7–2.5)
Age groups			
0–10	15.2 (9.3–21.3)	1	1
11–20	32.2 (20.7–43.7)	2.7 (1.3–5.4)	1.7 (0.6–4.4)
21–30	34.6 (23.6–45.7)	3 (1.7–5.2)	2.3 (0.8–6.4)
31–40	40.5 (30.2–50.8)	3.8 (2.1–6.9)	2.7 (0.7–10.0)
41–50	31.4 (22.9–40.0)	2.6 (1.7–4)	1.5 (0.5–4.6)
51–60	44.7 (34.7–54.8)	4.5 (2.6–7.9)	2.6 (0.9–6.8)
60<	32.0 (21.8–42.3)	2.6 (1.3–5.3)	1.3 (0.5–3.2)
Occupation			
Employed	36.1(26.4–45.7)	1	1
Unemployed	14.2 (–2.0–30.1)	0.3 (0.1–1.1)	0.2 (0.1–1)
Housewife	38.9 (31.3–46.5)	1.1 (0.6–2.02)	1.4 (0.6–3.2)
Retired	40.5 (21.4–59.6)	1.2 (0.5–2.8)	1.2 (0.3–4.7)
Student/Soldier	31.3 (20.3–42.2)	0.8 (0.5–1.5)	1.0 (0.4–2.7)
Children	9.1 (2.6–15.4)	0.2 (0.1–0.4)	0.3 (0.0–2.0)
Education			
Illiterate	35.7 (20.3–51.1)	1	1
First elementary school	33.1 (22.6–43.6)	1.0 (0.4–2.3)	1.0 (0.5–2.0)
Second elementary school	42.9 (28.8–56.9)	1.5 (0.6–4.2)	1.2 (0.4–3.8)
High school	35.7 (27.5–44.0)	1.0 (0.4–2.4)	0.8 (0.2–2.6)
University	31.7 (20.8–42.6)	1.0 (0.4–2.4)	0.6 (0.2–2.1)
Children under 6-yr	9.1 (2.5–15.6)	0.2 (0.0–0.6)	1.1 (0.2–7.4)
Water Source			
Tap water			
Yes	30.9 (26.4–35.5)	0.6 (0.4–1.2)	0.3 (0.0–0.9)
No	41.1 (25.2–57)		
Spring water			
Yes	38.1 (17.7–58.6)	2.1 (0.6–8.3)	0.7 (0.2–1.9)
No	32 (27.5–36.5)		
Filtered water			
Yes	29.9 (18.8–40.9)	1 (0.6–1.6)	0.3 (0.0–1.2)
No	33 (27.1–38.7)		
Travel history			
Yes	30.6 (24.1–37.1)	0.8 (0.5–1.3)	0.8 (0.4–1.5)
No	35.7 (26.6–44.7)		
Animal contact			
Yes	45.3 (28.8–61.7)	1.9 (1–3.6)	1.8 (1.0–3.2)
No	30.7 (25.9–35.5)		

a CI: Confidence interval;

b OR: Odds ratio;

c aOR: Adjusted odds ratio

Among possible risk factors investigated in this study, drinking water source and close animal contact were found to have a significant relationship with intestinal parasitic infections. The aOR for intestinal parasitic infections was 1.8 (95% CI 1.0–3.2) in animal-contact subjects. City tap water (aOR=0.3, 95% CI 0.0–0.9) intake showed a possible protective effect against intestinal parasitic infections. Intestinal parasitic infections showed no significant correlation with sex, age, occupation, education, or travel history.

## Discussion

The prevalence of both pathogenic and nonpathogenic intestinal parasites was high in residents of Roudehen. The drinking water source and close animal contact were the most significant factors in the infection of population. The results of the study showed the existence of several intestinal parasites of public health importance among residents of Roudehen. To our knowledge, this is the first well-designed study reporting the true prevalence of intestinal parasitic infections in the general population. The prevalence of enteric parasites in the residents of Roudehen was 32.7% (95 % CI 27.3–38), similar to a study (32.3%) conducted in apparently healthy rural and semirural inhabitants of western (Lorestan), northwestern (West Azerbaijan), and northeastern (Golestan) Iran (9). However, it is higher than the previous prevalence (19.3%) reported in the general population throughout the country (8) and lower than the prevalence (48.8%) reported in the rural areas of Bandar-Abbas (10). The variations in prevalence of intestinal parasites in these studies could be due mainly to the sensitivity of applied parasitological techniques as well as to diversity in socioeconomic, geographic, sanitary/hygiene, cultural, and educational status, and nutrition of study subjects. In this study, only a single case (0.2%) of helminth infection with *E. vermicularis* was detected. The high prevalence of protozoan parasites observed compared to helminths is in agreement with previous findings in Iran (6, 10, 11, 15) and other developing and developed countries (2, 16).

The prevalence of *Entamoeba* complex in the residents of Roudehen was (0.4%; 95% CI 0–1.1), similar to a study in Zahedan (0.5%) (7). However, it is lower than found in inhabitants of central, southern, and northern Iran (1.4%; 95% CI 1.2–1.5) (17), Bandar-Abbas (5.8%) (10), and Lorestan, West Azerbaijan, and Golestan (8.4%; 95% CI 6.9–10.3) (9). The pathogenic member of the *Entamoeba* complex, *E. histolytica,* was not found in the study population, and a single co-infection of *E. moshkovskii* with *E. dispar* was detected. Our study confirmed the previous finding showing extremely low prevalence of *E. histolytica* in the Iranian population. Haghighi et al. (7) and Solaymani-Mohammadi et al. (9) did not detect *E. histolytica,* and Hooshyar et al. found only eight *E. histolytica* infections among 101 successfully cultured *Entamoeba*-cyst positive isolates, resulting from screening 16592 individuals (18).

The most frequently detected intestinal protozoan was *Blastocystis sp.* (28.4%; 95% CI 23.7–33.0), similar to other studies in Iran (10, 11, 15) and in the world (19, 20). Epidemiological surveys have shown *Blastocystis* sp. to be the most prevalent human intestinal parasite worldwide (19, 20). As this parasite is transmitted to humans through the fecal-oral route, the rate of infection should be related to poor hygiene, consumption of contaminated food or water, and close animal contact (19, 21). Although the role of *Blastocystis* in disease is controversial (21, 22), it is a good criterion for hygiene.

In the present study, the prevalence of *Giardia* infection was 1.2% (95% CI 0.4–2.0), lower than the previous studies in Iran. The overall prevalence of *Giardia* has shown a significant declining trend during the past decade from 25.8% (15), 10.9% (8), 10.6% (9), 10.1% (7), 5.4% (11), and 2.5% (23), to 1.2% in this study. Beside of the improvement in general hygiene in the country, these variations are probably associated with the population and area of the diagnostic approaches used.

The true prevalence of *D. fragilis* in the healthy population is not clear, with literature reporting 0.2%–71% of gastrointestinal patients infected with *Dientamoeba* (24, 25). We found 1.1% (95% CI 0.2–2.0) of the apparently healthy population to harbor *Dientamoeba*, within the 0.5%–2.4% range reported in several studies in Iran (9, 26, 27).

In this study, several possible factors associated with intestinal parasites were investigated. Intestinal parasitic infections were more frequent in males at 33.9% (CI: 24.7–43.9) (aOR= 1.40, CI: 0.7–2.5), similar to other studies in Iran (11) and throughout the world (28). It is possible that greater participation by men in outdoor activities increases exposure to infection. The prevalence of parasitic infections in the age group 51–60 yr was 44.7% (95% CI 43.7–54.8) (aOR= 2.6, CI: 0.9–6.8), higher than other groups, similar to results of other studies (4, 16). The most common infections in this age group were *Blastocystis* sp (42.2%, 95% CI 31.5–52.9). This pattern may suggest that incidence of *Blastocystis* infection increases with age (28, 29). The prevalence of parasitic infection showed no significant relationship with occupation or education; although the highest prevalence was found in retired people at 40.5% (95% CI 21.4–59.6) (aOR= 1.3, 95% CI 0.3–5.5).

Household sanitation in this urban community was adequate. Few people consumed water from sources other than tap water. Prevalence of intestinal parasites in people who used spring water was 38.1% (95% CI 17.7–58.6), suggesting the possibility of waterborne transmission (aOR= 0.3, 95% CI 0.0–0.9). Close contact with animals was associated with the risk of intestinal parasitic infection of 45.3% (95% CI 28.8–61.7) (aOR= 1.9, 95% CI 1.0–3.2). The most prevalent parasite was *Blastocystis*. Considering the zoonotic nature of this parasite (19, 21, 22), infection by household animals, chiefly birds, dogs, and cats, seems probable.

The study design likely produced results that reflected the city population as a whole. The possible limitation was the low response rate (25.4%). For cultural reasons, collecting feces and passing it to others was difficult for people. However, with statically replacement of sample, a representative sample of the population was collected, and the findings of this study can be generalized to the Roudehen city population. This study was carried out with only urban residents, limiting generalization to the wider Iranian population; thus, further investigation in suburban and rural areas is needed. This study is the first of its type to report the prevalence of intestinal parasitic infections among residents in Roudehen city. We used a combination of conventional techniques (concentration and culture) for detecting enteric parasites and molecular procedure for distinguishing *Entamoeba* complex parasites. Further molecular studies for identification of *D. fragilis* are underway since its true prevalence is difficult to reveal by conventional method.

## Conclusion

The present study revealed a high prevalence of enteric protozoan parasite infection among citizens of Roudehen in Iran. Protozoan infections were more common than helminth infections. The neglected intestinal parasite *Blastocystis* was recognized as one of the most significant causes of infection. Consuming tap water had a possible protective effect against intestinal parasites, and contact with animals posed a risk for infection. As most of the detected parasites are transmitted via a water-resistant cyst, public and individual education on personal hygiene should be considered to improve health and prevent transmission of intestinal parasites to people living in this city.
